# Botulinum Toxin Injections into the Masseter and Temporalis Muscles vs. Oral Methocarbamol for Temporomandibular Myofascial Pain in Adults: A Randomized Clinical Trial

**DOI:** 10.3390/healthcare14213630

**Published:** 2026-10-26

**Authors:** Celina García-Pontigo, Antonio Francisco Ferrández-Martínez, Raquel Alarcón-Rodríguez, Fernando Espín-Gálvez, Mario Paños-Lorenzo, Ana Rocío García-Carricondo

**Affiliations:** 1 Oral and Maxillofacial Surgery, Hospital Universitario Torrecárdenas, 04009 Almería, Spain; antoniofm95@hotmail.com (A.F.F.-M.);; 2 Department of Nursing, Physiotherapy and Medicine, Faculty of Health Sciences, University of Almería, 04120 Almería, Spain; ralarcon@ual.es; 3 Research Group CTS-1127 “Epidemiology and Public Health”, University of Almería, 04120 Almería, Spain; 4 Health Research Center (CEINSA), University of Almería, 04120 Almería, Spain

**Keywords:** botulinum toxin type A, methocarbamol, muscle relaxants, myofascial pain syndrome, temporomandibular disorders, temporomandibular joint, masticatory muscles, bruxism, temporalis muscle, trigger points

## Abstract

**Highlights:**

**What are the main findings?**

- Both BTX-A and oral methocarbamol were associated with significant within-group reductions in overall pain intensity (VAS) and improvements in maximum mouth opening, with no statistically significant between-group differences observed at five weeks.
- In an exploratory muscle-by-muscle analysis without adjustment for multiple comparisons, BTX-A was associated with a statistically significant greater reduction in active trigger points in the right masseter and right temporalis muscles compared with methocarbamol; no differences were observed for the left-sided muscles.

**What is the implication of the main findings?**

- Single-dose BTX-A was not superior to a 5-week oral methocarbamol regimen for overall pain intensity or mandibular mobility. The side-specific reduction in right-sided trigger points should be considered exploratory and hypothesis-generating, requiring confirmation in studies with formal adjustment for multiple testing.

**Abstract:**

**Background/Objectives:** Treatment of temporomandibular myofascial pain includes physical therapy, the use of occlusal splints, pharmacological management, and botulinum toxin type A (BTX-A) injections. The aim of this clinical trial was to determine whether BTX-A provides greater clinical benefit than methocarbamol in the management of myofascial pain. **Methods:** A randomized clinical trial with two parallel groups was conducted, including 235 patients: control group (methocarbamol, *n* = 111) and intervention group (BTX-A, *n* = 124). Pain perception was assessed through the number of trigger points detected in the masseter and temporalis muscles, the McGill Pain Questionnaire, and the visual analog scale (VAS). Oral opening was measured using the TheraBite Range-Of-Motion scale in millimeters. **Results:** Both groups showed significant within-group decreases in overall pain intensity (VAS) and increases in maximum mouth opening (*p* < 0.001). Baseline-adjusted comparisons at five weeks showed no significant between-group differences in pain (VAS: *p* = 0.130) or maximum mouth opening (TheraBite: *p* = 0.125). In an exploratory analysis by muscle site, without correction for multiple comparisons, the BTX-A group showed a greater reduction in active trigger points in the right masseter (*p* = 0.028) and right temporalis (*p* = 0.009) but not in the left-sided muscles (*p* > 0.05). **Conclusions:** Both BTX-A and methocarbamol were associated with significant improvements in overall pain intensity and maximum mouth opening, with no statistically significant between-group differences at five weeks. The greater reduction in right-sided trigger points observed with BTX-A is an exploratory finding that may reflect multiplicity and requires confirmation.

## 1. Introduction

Myofascial and temporomandibular pathology is the most common disorder affecting the temporomandibular joint (TMJ) [1]. This pathology is more prevalent among women, with adults between 18 and 60 years being the most frequently affected age group [2]. It significantly impacts the quality of life of affected patients [3,4].

The term myofascial pain refers to a broad range of discomforts primarily related to soft tissues [5]. Its etiology is multifactorial, with biological, psychological, behavioral, and clinical factors potentially contributing to its onset and persistence [6,7]. Clinical manifestations may include masticatory muscle pain, painful or tender points, headache, and impaired mandibular function [5,8].

Temporomandibular myofascial pain is a condition defined by trigger points that elicit a nociceptive response and a significant increase in muscle tone, resulting in painful hypertrophy. Its origin is multifactorial, combining autonomic factors, central sensitivity, or even psychological factors. Patients experience spontaneous pain upon mastication or palpation in the periarticular region, often accompanied by sudden spasms in the masticatory muscles (pterygoid, temporalis, and masseter muscles) [5,8,9].

The management of temporomandibular disorders encompasses a wide range of therapeutic modalities and therefore requires a multidisciplinary approach tailored to the individual patient’s clinical presentation. Over the years, a wide range of therapeutic approaches have been investigated, including lifestyle modifications, pharmacological therapy, physiotherapy, and occlusal splint therapy [5,10]. Muscle relaxants such as methocarbamol and diazepam are commonly used, though their limitations include adverse effects such as drowsiness. More invasive therapies, such as injections with anesthetics or muscle relaxants, arthrocentesis, arthroscopy, and even open surgery, are selected in more severe cases with variable results [10].

Occlusal splints remain one of the most widely prescribed conservative treatments for patients with temporomandibular disorders and sleep or awake bruxism owing to their widespread clinical use and favorable safety profile. Beyond protecting the dentition against excessive occlusal forces and tooth wear associated with bruxism, these appliances are intended to reduce functional overload of the masticatory system and promote neuromuscular relaxation. While current evidence suggests that occlusal splints may improve pain and mandibular function in selected patients, particularly those with myogenous temporomandibular disorders, their superiority over other conservative interventions remains uncertain. Nevertheless, because of their non-invasive nature and potential to preserve dental structures while contributing to symptom control, occlusal splints continue to be recommended as a first-line therapeutic option in routine clinical practice [5,10].

Botulinum toxin type A (BTX-A) has emerged as an option for managing myofascial pain and migraine-like symptoms. However, its efficacy remains controversial due to the inconsistent results across the limited existing studies. Some of those show a lack of significant symptom improvement [11,12], whereas more recent randomized, double-blind, placebo-controlled evidence has demonstrated reductions in orofacial pain, headache intensity and frequency, and tender points following BTX-A treatment, with a significant between-group reduction in the number of tender points compared with saline placebo [8]. Additional studies report not just a significant pain reduction in the masseter region but also decreased analgesic requirements during the study period [13]. Meta-analytic evidence has also demonstrated significant reductions in pain intensity with BTX-A in patients with muscular temporomandibular disorders [14].

Botulinum toxin type A (BTX-A) induces reversible paralysis of the masseter and temporalis muscles following a single injection. However, repeated administrations have been associated with histological and functional changes that may persist over time [15,16,17]. Current evidence distinguishes the transient effects observed after a single administration from the more pronounced structural alterations reported following repeated injections, even if they are inconsistent [15,18].

Experimental studies have suggested that repeated BTX-A injections may somehow be associated with muscle atrophy, altered expression of atrophy-related molecular markers (Atrogin-1/MAFbx and MuRF-1) [19,20], impaired autophagic activity, and reduced desmin expression, suggesting a limited regenerative capacity of the affected muscle [21]. These structural changes are accompanied by functional alterations, including reduced bite force, decreased muscle thickness and contractility, and impaired masticatory performance [15]. Compensatory adaptations in adjacent masticatory muscles have also been pointed out, potentially affecting normal mandibular biomechanics [15,22].

Overall, the available evidence suggests that the effects of a single BTX-A administration are largely reversible, whereas repeated injections may increase the risk of persistent muscle atrophy, bone remodeling, and long-term functional impairment [23]. Therefore, repeated treatment should be considered cautiously, balancing its clinical benefits against the potential risk of sustained morphological and functional changes [24].

This clinical trial was specifically designed to compare two pain management strategies—systemic pharmacological therapy and botulinum toxin type A (BTX-A)—in patients with persistent temporomandibular myofascial pain requiring additional therapeutic intervention.

## 2. Materials and Methods

### 2.1. Study Design

A two-arm parallel randomized controlled clinical trial was conducted, in which the interventions consisted of botulinum toxin type A (BTX-A, BOTOX®, Allergan, Inc., Irvine, CA, USA) injections or, on the other hand, oral muscle relaxant therapy.

Although current international guidelines recommend conservative non-pharmacological management as the first-line treatment for temporomandibular myofascial pain, methocarbamol (Robaxin®, Almirall, S.A., Barcelona, Spain) is routinely used in our clinical practice for symptomatic treatment of acute and subacute myalgia because of its favorable safety profile. Therefore, it was selected as the active comparator to assess whether local treatment with botulinum toxin type A (BTX-A) offered greater clinical benefits than systemic pharmacological therapy.

The study included adult patients diagnosed with temporomandibular myofascial pain according to the Diagnostic Criteria for Temporomandibular Disorders (DC/TMD) [25] and the trial was reported in accordance with the CONSORT 2010 guidelines [26].

### 2.2. Study Population

Participants were recruited by Oral and Maxillofacial Surgery (OMFS) attendings from Torrecárdenas Hospital (Almería, Spain) between January 2022 and December 2023. Eligible participants attending the outpatient clinic were consecutively recruited according to predefined inclusion criteria.

All participants had previously received conservative treatment, including physiotherapy and occlusal splint therapy. However, these interventions had failed to provide adequate symptom control, and patients continued to report persistent temporomandibular myofascial pain. Consequently, to ensure treatment homogeneity and minimize potential confounding factors, all ongoing conservative therapies were discontinued before study enrollment, allowing the effects of the assigned intervention to be evaluated independently.

**Inclusion criteria** involved ages between 18 and 65 years and a confirmed diagnosis of temporomandibular myofascial pain according to the Diagnostic Criteria for Temporomandibular Disorders (DC/TMD) following the validated Axis I diagnostic algorithm for myalgia/myofascial pain [25]. Participation was voluntary, and written informed consent was obtained from all participants.

**Exclusion criteria** included a history of locoregional radiotherapy, cognitive impairment or language barriers that could interfere with proper assessment, previous facial fractures, or prior open or arthroscopic temporomandibular joint surgery related to tumor management. Patients diagnosed with neoplastic or rheumatologic diseases affecting the parotid gland or the temporomandibular joint areas were excluded as well. Medical history of trigeminal neuralgia, pregnant women, individuals without the required imaging studies, or those with evidence of infectious temporomandibular joint disease were also excluded. Those patients diagnosed with generalized chronic pain syndromes (fibromyalgia) or systemic neuromuscular disorders were also excluded to ensure a homogeneous sample of localized temporomandibular myofascial pain.

All participants underwent radiological assessment including magnetic resonance imaging (MRI) to exclude structural temporomandibular joint pathology or abnormalities and panoramic radiography to rule out other pathological conditions involving the maxilla or mandible.

Treatment discontinuation or withdrawal from the study occurred in cases of allergic reactions, drug intolerance, cutaneous reactions, adverse events, pregnancy, trauma, or injury. Withdrawal was also permitted at any time upon revocation of informed consent.

### 2.3. Patient Recruitment and Randomization

The sample size was determined using the G*Power software (version 3.1.9.7, Heinrich-Heine-Universität Düsseldorf, Düsseldorf, Germany) for repeated-measures ANOVA across factors, with the variables “painful trigger points”, VAS scale, subjective pain perception (assessed via Pain Perception Index, PPI), and oral opening. A 5% level of significance and a power of 80% were considered, establishing a minimum sample size of 111 patients per group of treatment [27]. Recruitment continued until this minimum was reached in both groups.

Potential candidates presenting with temporomandibular myofascial pain were randomly allocated, after verifying the inclusion and exclusion criteria, with equal probability (1:1 allocation ratio) to either the control group (oral methocarbamol) or the experimental group (BTX-A) using simple randomization (without blocking or stratification) based on Epidat 4.2 (Consellería de Sanidade, Xunta de Galicia, Santiago de Compostela, Spain) software-generated random numbers. Recruitment and enrollment were conducted by attending oral and maxillofacial surgeons. In accordance with CONSORT transparency guidelines, we explicitly state that no separate allocation concealment mechanism was employed. As expected with simple randomization, this procedure did not guarantee equal group sizes, resulting in 111 patients in the control group and 124 in the BTX-A group.

Due to the distinct administration routes of the evaluated interventions, a single intramuscular injection session of BTX-A versus a 5-week continuous oral regimen of methocarbamol, complete patient blinding was not feasible without a double-dummy design involving sham injections or placebo tablets. Therefore, the trial was conducted under an open-label, active-controlled design. To reduce measurement variability, all outcome evaluations, trigger point verifications, and mandibular mobility measurements were performed by calibrated oral and maxillofacial surgeons using standardized Case Report Forms (CRFs) and objective measurement instruments (TheraBite^®^ ROM scale, Atos Medical Inc., New Berlin, WI, USA) in strict adherence to the Diagnostic Criteria for Temporomandibular Disorders (DC/TMD) protocol.

### 2.4. Outcome Measures

The study sample consisted of adult patients aged between 18 and 65 years diagnosed with temporomandibular myofascial pain. No participant showed radiological evidence of temporomandibular joint abnormalities or other lesions or diseases involving the mandible or maxilla, as confirmed by panoramic radiography and magnetic resonance imaging (MRI). In addition, all subjects voluntarily agreed to participate and provided written informed consent prior to inclusion in the study.

**Pain progression** was evaluated using two variables: the number of trigger points in the masseter and temporalis muscles and the VAS scale. A standardized Case Report Form (CRF) was used for data collection throughout this study.

**Figure 1:**
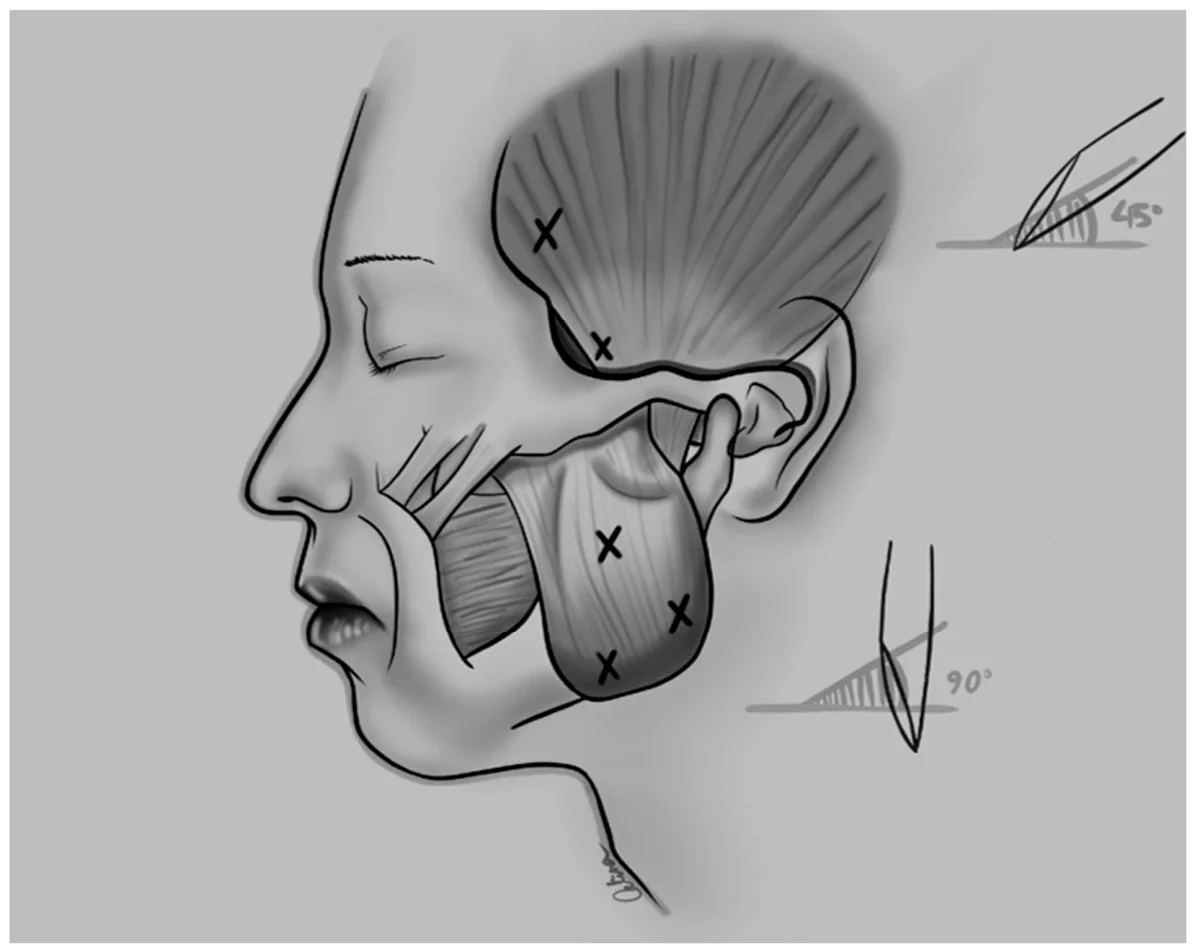
BTX-A trigger points and infiltration dots in temporalis and masseter muscles.

As part of the baseline assessment, a visual and quantitative pain mapping component was incorporated. Patients performed a self-assessment by marking the anatomical locations of their perceived pain on a standardized reference image (Figure 1). This approach allowed the systematic documentation of pain distribution before the clinical examination and facilitated comparison with the findings obtained during the standardized DC/TMD assessment.

The number of trigger points was quantified on a 0 to 3 scale according to the number of painful areas reported by the patients within each of the right and left masseter and temporalis muscles, with 0 indicating the absence of active trigger points in that muscle. These self-reported painful sites were subsequently verified through standardized manual palpation performed by calibrated oral and maxillofacial surgeons in accordance with the Diagnostic Criteria for Temporomandibular Disorders (DC/TMD). Only trigger points reproducing the patient’s familiar pain during clinical examination were included in the final analysis.

General pain perception was evaluated using the visual analog scale (VAS), which consists of a 10 cm straight line where the patient marked an “X” at the point that best represented their pain perception at that moment, by indicating on a numbered scale from 0 to 10, 0 being “no pain” and 10 being “maximum pain” [28]. This scale has been validated for pain measurement based on Cronbach’s alpha coefficient, which determines that the closer the value is to 1, the greater the reliability of the measurement instrument. According to the study by Kersten, White, and Tennant, this scale presents a Cronbach’s alpha coefficient ranging from 0.88 to 0.97, indicating a reliable and precise quantification method [28].

Subjective pain perception was also assessed on a 0 to 5 scale using the McGill Pain Questionnaire, where the score was determined based on five descriptive categories arranged in increasing pain intensity [29,30]. According to the study by Dworkin, Turk, and Revicki et al., this scale has a Cronbach’s alpha coefficient ranging from 0.86 to 0.95, making it a highly precise and effective pain assessment method [30].

Oral opening was measured using the TheraBite Range-Of-Motion (ROM)^®^ device, which provided a millimeter-scale assessment of maximum mouth opening capacity.

Clinical examinations and measurements of maximum mouth opening were performed by experienced oral and maxillofacial surgeons at the hospital. The clinical examination included systematic manual palpation of the masticatory muscles to reproduce and confirm the patients’ familiar pain and the trigger points documented in the case report form (CRF), in accordance with the Diagnostic Criteria for Temporomandibular Disorders (DC/TMD) [25].

To minimize inter-examiner variability, all participating oral and maxillofacial surgeons underwent prior calibration in the application of the DC/TMD protocol and in the use of the TheraBite^®^ Range of Motion Scale as well [25].

To ensure methodological consistency and reduce measurement variability, all participants were evaluated using the same standardized Case Report Form (CRF), the same anatomical reference image (Figure 1), and the same 0 to 3 trigger point scoring system at baseline and at the five-week follow-up. Identical assessment procedures were applied at both time points, with all clinical examinations performed according to the Diagnostic Criteria for Temporomandibular Disorders (DC/TMD) [25].

### 2.5. Intervention

Patients in the Control Group received pharmacological treatment with orally administered muscle relaxants (methocarbamol 500 mg, at a dose of 2 pills every 6 h) for five weeks.

The 5-week follow-up period was selected based on the considerable heterogeneity observed among previous studies regarding follow-up intervals, treatment protocols, injection sites, doses, and diagnostic criteria [31,32]. The available evidence suggests that the analgesic effect of BTX-A may become clinically apparent within the first weeks after injection, with a few studies reporting relevant changes between 2 and 6 weeks, although the magnitude and timing of this response remain inconsistent [15]. Therefore, a 5-week assessment was considered an appropriate intermediate time point to capture the early clinical response to BTX-A, allowing sufficient time for its initial therapeutic effects to develop in those cases where it actually took place.

In the Intervention Group, patients underwent botulinum toxin type A (BTX-A, BOTOX^®^, Allergan) infiltration while seated, with the occlusal plane parallel to the floor, following proper aseptic preparation of the area. The infiltration began with the temporalis muscle, starting the procedure in the anterior region by slowly introducing the substance with the needle positioned at an approximately 45º inclination relative to the skin surface. When the muscle was reached, the infiltration was administered directly. Two separate injections were performed at equidistant points to ensure complete distribution within the muscle. Each injection contained 10 U, reaching a total of 20 U per muscle.

Subsequently, infiltration of the masseter muscle was performed, starting slowly from the anterior region and proceeding until full infiltration of the muscle was achieved. In this case, the needle was positioned perpendicularly to the skin surface, and a total of three injections were administered, each containing 10 U, summing up to a total dose of 30 U per muscle [11,33,34].

All measurements of pain perception and oral opening were recorded prior to treatment initiation and five weeks after administration.

### 2.6. Procedure

Patients completed an initial pain assessment using a Case Report Form (CRF), which included the following items: an image in which they marked the number (0, 1, 2, or 3) and location of the trigger points perceived in the right and left masseter and temporalis muscles; the McGill Pain Questionnaire, where they selected a score between 0 and 5 corresponding to the adjective that best described their pain perception; and the Visual Analog Scale (VAS) ranging from 0 to 10. Subsequently, the oral opening was measured by the surgeon using the TheraBite Jaw ROM Scale Pack^®^.

Patient follow-up was conducted five weeks after the infiltration in the Intervention Group (botulinum toxin type A-BTX-A) and after the equivalent pharmacological treatment period in the Control Group (methocarbamol). The establishment of this specific observation period aimed to evaluate the early effectiveness of botulinum toxin (BTX-A) treatment, as there is currently no standard protocol that defines an exact point in time for symptom improvement. This lack of standardization is evident in the variability across studies conducted to date, where the evaluation period for symptom relief typically ranges between 2 and 4 weeks post-treatment [12,33,34,35].

Potential adverse events, including allergic reactions, drug intolerance, and cutaneous reactions, were monitored during follow-up visits and recorded when reported or clinically identified. Other relevant events, such as pregnancy, trauma, or injury, were also documented during follow-up. No treatment-related adverse events were reported or identified during the follow-up period.

### 2.7. Ethical Considerations

The study protocol received approval from the Research Ethics Committee of Almería (Research Ethics Committee of Hospital Universitario Torrecárdenas), Spain (FEG-CGP-2020-04) on 24 February 2021, and the trial was registered in advance with the European Union Drug Regulating Authorities Clinical Trials Database (Eudra-CT number 2020-004661-39) on 1 February 2021. All procedures were conducted in accordance with the ethical principles of the Declaration of Helsinki [36]. Prior to participation, all patients provided informed consent, both verbally and in writing, with guarantees of confidentiality and data protection.

### 2.8. Statistical Analysis

Statistical analysis was performed using SPSS software (version 30.0, IBM Corp., Armonk, NY, USA). Descriptive statistics were computed, presenting qualitative variables as absolute frequencies and percentages, and quantitative variables as means and standard deviations (SD). The normality of the data distribution was assessed using the Kolmogorov–Smirnov test. To evaluate treatment efficacy, both within-group (intragroup) and between-group (intergroup) analyses were conducted. For within-group longitudinal comparisons (from baseline to the five-week follow-up), paired Student’s t-tests were employed for normally distributed quantitative variables. To compare clinical outcomes between the two treatment groups (BTX-A vs. Methocarbamol) while accounting for baseline variability, an Analysis of Covariance (ANCOVA) was performed utilizing the post-treatment score as the dependent variable and the baseline score as the covariate.

For the analysis of localized active trigger points, a two-way mixed-design Analysis of Variance (ANOVA) was performed using SPSS software (version 30.0), with one within-subject factor (Time: baseline vs. 5 weeks) and one between-subject factor (Group: BTX-A vs. Methocarbamol) to evaluate the statistical significance of the Group × Time interaction. Statistical significance was set a priori at *p* < 0.05.

## 3. Results

### 3.1. Participant Flow and Baseline Characteristics

**Figure 2:**
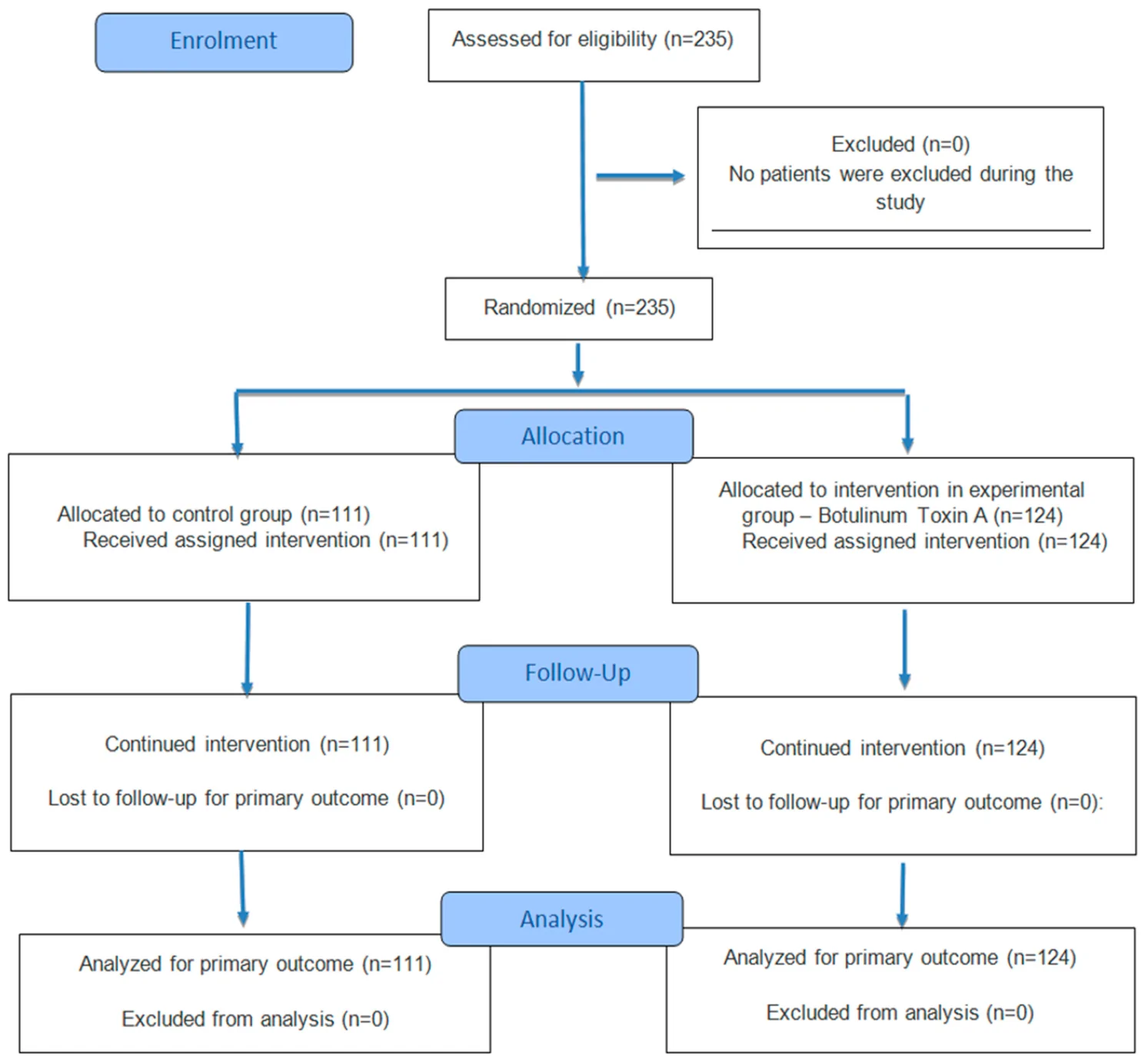
CONSORT flow diagram of the phases of a randomized parallel-group clinical trial.

A total of 235 patients with confirmed temporomandibular myofascial pain were included (Control *n* = 111; BTX-A *n* = 124), and all of them received their assigned treatment with complete follow-up (Figure 2).

Baseline demographic and clinical characteristics were homogeneous between cohorts, with no statistically significant differences (Table 1).

### 3.2. Active Trigger Points

With respect to the evaluation of active temporomandibular myofascial trigger points, intragroup analysis revealed that both treatment groups (BTX-A group and methocarbamol group) showed statistically significant reductions in the number of active temporomandibular myofascial trigger points across all evaluated muscles after five weeks of follow-up.

In the right masseter, trigger points decreased from 2.18 (0.90) to 1.66 (1.01) in the methocarbamol group, and from 1.89 (1.11) to 1.37 (0.99) in the BTX-A group. The two-way mixed-design ANOVA on raw individual patient data demonstrated a statistically significant Group × Time interaction effect favoring the BTX-A group (*F* = 4.88, *p* = 0.028). Similarly, in the right temporalis, the control group showed a reduction from 1.86 (0.95) to 1.33 (0.98), whereas the BTX-A group exhibited a decrease from 1.60 (1.17) to 0.99 (0.58), yielding a significant interaction (*F* = 6.95, *p* = 0.009). No statistically significant interaction effects were observed for the left-sided musculature (*p* = 0.601 and *p* = 0.132, respectively) (Table 2).

### 3.3. Pain Intensity and Pain Perception

Longitudinal within-group analysis revealed significant reductions in pain levels from baseline to the 5-week follow-up in both treatment cohorts (*p* < 0.001). Within the intervention group (BTX-A), the mean VAS score decreased by an average of 2.50 points, falling from 7.54 (1.71) at baseline to 5.04 (2.62) at five weeks. Similarly, the control group (methocarbamol) exhibited an average reduction of 2.03 points, decreasing from a baseline score of 7.44 (1.62) to 5.41 (2.75) at the end of the follow-up period (Table 3).

The baseline-adjusted between-group comparison of post-treatment pain scores using ANCOVA showed no statistically significant difference between the BTX-A and methocarbamol groups at five weeks (*p* = 0.130).

Regarding the PPI, both groups showed significant improvements over time (*p* < 0.001). The mean PPI score in the BTX-A group decreased from 3.34 (1.16) at baseline to 2.08 (1.12) at five weeks, while the methocarbamol group decreased from 3.32 (1.05) to 2.35 (1.15). The baseline-adjusted intergroup comparison (ANCOVA) at five weeks showed no statistically significant difference between groups (*p* = 0.071) (Table 3).

### 3.4. Maximum Mouth Opening

Mandibular mobility, evaluated using the TheraBite Range-Of-Motion device, showed relevant within-group improvements from baseline to the 5-week follow-up in both treatment groups. In the control group (methocarbamol), the mean maximum mouth opening increased from 37.91 (4.52) mm to 38.89 (3.28) mm. Meanwhile, the intervention group (BTX-A) demonstrated an increase from a baseline of 36.90 (5.76) mm to 39.02 (3.03) mm in five weeks (Table 4).

Nevertheless, when comparing post-treatment mouth opening values between the two groups, the difference in maximum mouth opening between the BTX-A and methocarbamol groups at five weeks was not statistically significant (*p* = 0.125).

## 4. Discussion

In the present study, a randomized clinical trial was conducted to evaluate two therapeutic strategies for temporomandibular myofascial pain: oral muscle relaxant therapy (methocarbamol) and intramuscular botulinum toxin type A (BTX-A) injections. The objective was to observe clinical outcomes regarding pain perception, trigger points, and mandibular mobility under standardized clinical conditions.

The findings of the current study indicate that both BTX-A and systemic pharmacological therapy were associated with reductions in temporomandibular myofascial pain and improvements in maximum mouth opening, with no statistically significant between-group differences observed for these outcomes, except for the number of trigger points in the right masseter and temporalis muscles, an exploratory finding discussed below.

The application of botulinum toxin type A (BTX-A) for orofacial pain remains controversial due to the heterogeneity of the papers, although some of them are showing promising results. When reviewing published articles, the research teams of Kim SR et al. [8], Chaurand [31], Guarda-Nardini [35], Kurtoglu [33], and Patel [37] all demonstrated a reduction in pain in those treated with botulinum toxin A (BTX-A) compared with control groups. However, significant differences among these and other studies may affect the homogeneity of the results. Conversely, studies conducted by Reeve [12], Minston [11], and De Carli [38] did not observe significant differences compared with placebo or other treatment modalities.

Regarding maximum mouth opening, the literature also revealed variable results. While Guarda-Nardini [35] reported an increase in mouth opening, Reeve et al. [12] found no significant improvement in maximal incisal opening from baseline within either the BTX-A or placebo group, and no significant difference was observed between the two groups. Similarly, Minston et al. [11] observed no significant between-group differences in maximum jaw opening.

A noteworthy bias in the aforementioned studies, which was mitigated in the present trial, is the prior administration of treatments that could alter the effectiveness of the drugs employed [8,25].

Our study included a considerably larger number of participants than most previous trials on this topic, such as those by Reeve et al. [12], Kurtoglu et al. [33] and Guarda-Nardini et al. [35], which increases the precision of the estimated treatment effects.

However, a larger sample size does not by itself reduce sex-related selection bias. As in previous trials, such as Minston et al., in which women represented 83% of the BTX-A group and 91% of the placebo group [11], and Kurtoglu et al., in which 20 of 24 participants were women [33], the present sample showed a marked female predominance (83%). This distribution is consistent with the higher prevalence of temporomandibular disorders among women [2] and is therefore likely to reflect the clinical population seeking treatment rather than a recruitment artifact. Nevertheless, our findings are mainly generalizable to female patients, and their applicability to men should be interpreted with caution. In addition, no sex-stratified analysis was performed, so potential sex-related differences in treatment response could not be explored and should be addressed in future studies.

In the exploratory muscle-by-muscle analysis, we observed a statistically significant reduction in the number of active trigger points located in the right temporalis and right masseter muscles of the intervention group. This outcome aligns with studies such as the one published in The Journal of Headache and Pain in 2016 by Pihut et al. [13], which evaluated the efficacy of botulinum toxin type A (BTX-A) infiltration in temporomandibular myofascial pain, primarily affecting the masseters. That study demonstrated statistically significant effectiveness of botulinum toxin type A (BTX-A) in reducing tension headache and pain. Similar results were obtained in Guarda-Nardini’s study, which compared two populations—one receiving botulinum toxin with marked improvement and the other administered a saline solution—showing no change in pain assessment [35].

Another notable discrepancy among studies is the dosage of the drug administered. Ranges vary from conservative doses of 60 U per hemiface, as in Chaurand’s study [31], to 100 U evenly distributed between both masseter and temporalis muscles (50 U per side, infiltrating 20 U in each temporalis and 30 U in each masseter), as presented in Guarda-Nardini’s study [35] and in our trial. Higher doses, such as the 200 U used in Kim et al. study [8], have also been reported. The absence of a standardized treatment protocol for the use of botulinum toxin type A (BTX-A) in temporomandibular myofascial pain syndromes results in this variability in dosage selection.

Additionally, there are significant differences in patient monitoring schedules. Our study conducted follow-ups five weeks after starting treatment to assess early pharmacological responses. However, these tracking periods widely differ in previous studies, ranging from 14 days post-infiltration (Kurtoglu et al. [33]) to serial evaluations at one week, one month, and six months (Guarda-Nardini [35]). Different schedules such as three weeks (Kim et al. [8]) or four weeks (Chaurand [31]) have also been reported.

Regarding trigger point reduction, a statistically significant Group × Time interaction favoring the BTX-A group was observed exclusively in the right masseter and right temporalis muscles. Rather than interpreting this unilateral finding as a definitive anatomical or biomechanical property of the masticatory apparatus, it must be interpreted with caution. Performing multiple statistical comparisons across individual muscle sites increases the probability of Type I error, and this side-specific result may represent a multiplicity artifact rather than a true biological asymmetry. Unless confirmed in future studies employing formal adjustment for multiple testing, this observation should be considered purely exploratory.

Taken together, no statistically significant differences between BTX-A and methocarbamol were found in the main clinical outcomes, including overall pain intensity, subjective pain perception, and maximum mouth opening. The greater reduction in active trigger points observed in the right masseter and temporalis muscles should be considered an exploratory, hypothesis-generating finding and does not, by itself, establish a localized benefit of BTX-A.

The outcomes of our study suggest that BTX-A may provide a localized benefit by reducing the number of active trigger points in the right masseter and temporalis muscles, while its effect on global clinical outcomes was comparable to that of methocarbamol, although this localized effect should be regarded as exploratory.

Additionally, a thorough evaluation of congenital or acquired muscular alterations, motor dysfunctions, and baseline nocturnal resting habits is necessary, as these factors may distort the assessment of drug effectiveness in temporomandibular myofascial pain treatment [3,4].

The practical implications of this study are significant: according to Alsahman et al. [4], patients with TMJ pain experience a reduced quality of life, largely due to the impact of their symptoms on social functioning. Although quality of life was not directly assessed in the present trial, previous literature suggests that effective symptomatic management of temporomandibular pain may improve daily functioning [4]. Nevertheless, the highly multifactorial nature of temporomandibular myofascial pain makes it difficult to establish a single pharmacological protocol as an effective treatment [3,10].

Another important aspect to consider is the potential medium- and long-term effects of botulinum toxin type A (BTX-A) [17,22,39]. Although several studies have reported reductions in temporomandibular myofascial pain, many of them have also reported transient decreases in muscle thickness, muscle activity, and bite force following injection, together with possible adaptive changes in the mandibular bone after repeated administrations [15,21,32].

However, current evidence remains limited and heterogeneous, and the long-term clinical relevance and reversibility of these findings have not yet been fully established [17,22,39]. Therefore, while BTX-A may be a reasonable therapeutic option when used appropriately, repeated administrations should be approached with caution until further high-quality longitudinal studies become available [16,40,41].

Beyond short-term symptomatic relief, the clinical utilization of BTX-A warrants a critical evaluation of its histological, molecular, and functional consequences on the masticatory system. Intramuscular injection of BTX-A induces a temporary functional denervation of the masseter and temporalis muscles by blocking acetylcholine release [16,17]. At the molecular level, this induced muscle hypofunction triggers a rapid upregulation of muscle-specific ubiquitin ligases, specifically Atrogin-1 (MAFbx) and MuRF-1, which accelerate proteasomal degradation and muscle fiber atrophy [19,20].

Histologically, recent evidence indicates that BTX-A-induced masseter atrophy is closely associated with impaired autophagic flux and disrupted mitophagy within the muscle tissue, without necessarily triggering apoptosis [19,20,23]. Additionally, a localized reduction in desmin expression has been reported, suggesting a compromised structural regenerative capacity of the affected muscle fibers [15,16,17]. These microstructural alterations clinically translate into transient decreases in muscle thickness, contractility, and maximum bite force [4]. Furthermore, unilateral muscular hypofunction may induce compensatory neuromuscular and hypertrophic adaptations in adjacent or contralateral masticatory muscles, potentially altering normal mandibular biomechanics and leading to localized bone remodeling or bone resorption at the mandibular condyle [16,17,39].

While the cellular and structural alterations following a single BTX-A administration appear to be largely reversible over time, repeated or long-term therapeutic cycles may increase the risk of persistent muscle atrophy, joint instability, and permanent musculoskeletal remodeling [16,17,39]. In the present trial, we evaluated only a single BTX-A administration over a five-week follow-up period. This short-term observation window represents a limitation of our study, as it prevents drawing definitive conclusions regarding long-term tissue recovery or the cumulative safety profile of repeated injections.

To establish more robust clinical guidelines, future randomized trials should incorporate extended follow-up periods (6 to 12 months) combined with objective diagnostic tools to monitor both long-term therapeutic sustainability and structural safety.

The selection of a five-week follow-up was based on the current heterogeneity of the available literature, as no consensus has been reached regarding the most appropriate time point for assessing the clinical effects of BTX-A in myogenous temporomandibular disorders. Published studies have employed widely varying follow-up periods and have reported inconsistent outcomes, with some demonstrating early improvements within the first month and others suggesting that the full therapeutic effect may only become apparent after several months [8,31,32,35]. This variability highlights the absence of a standardized follow-up protocol and supports the rationale for the five-week assessment used in the present clinical trial.

In addition, the design of the present study warrants further consideration. A potential limitation of this clinical trial is the absence of a placebo injection group, which may have limited complete blinding and cannot entirely exclude a placebo effect associated with the injection procedure itself.

However, this decision was made deliberately to avoid exposing participants in the control group to an invasive procedure without an expected therapeutic benefit. Although saline injections have been used as placebo controls in previous studies, they still require needle penetration and may be associated with discomfort and procedure-related adverse events. Since the primary objective of the present trial was to compare BTX-A with a routinely prescribed pharmacological treatment in our clinical practice, we considered an active pharmacological comparator to be a more appropriate and ethically justified control.

Moreover, patient-reported outcomes such as pain perception may be influenced by placebo responses. Maximum mouth opening was measured in millimeters using a standardized instrument (TheraBite^®^), which improves measurement reproducibility; however, it depends on patient effort and was recorded by non-blinded assessors, so it is not free of expectancy-related bias either. Therefore, this quantitative measure complements, but does not replace, the subjective pain assessments, and all outcomes should be interpreted in light of the open-label design [17,22,39].

Several methodological limitations of the present trial must be acknowledged. The trial lacked a formal allocation concealment mechanism and outcome assessor blinding, introducing a potential risk of selection, detection, and expectancy bias. A baseline imbalance was present in the right masseter trigger point count, suggesting that regression to the mean may have partially influenced the observed longitudinal change. Performing multiple statistical comparisons across individual muscle sites without formal adjustment for multiple testing increases the probability of Type I error; thus, the unilateral significance observed on the right side should be considered an exploratory finding rather than a definitive biological asymmetry.

Furthermore, our assessment relied primarily on patient-reported pain scales (VAS and McGill Pain Questionnaire) and examiner-dependent trigger point palpation, with maximum mouth opening being the only quantitative functional parameter. Although the pain scales used are validated instruments with high internal consistency, objective physiological measures, such as surface electromyography of the masticatory muscles, maximum bite force, pressure algometry, and masticatory performance tests, were not recorded. The inclusion of these objective parameters would have provided stronger, quantitative evidence regarding the functional impact of both therapies, and their absence represents a limitation that should be prioritized in future trials [8,33,35].

Finally, safety outcomes were not assessed using a systematic or predefined adverse-event monitoring protocol. Although no treatment-related adverse events were reported or clinically identified during follow-up, the non-systematic nature of adverse-event monitoring limits the conclusions that can be drawn regarding the comparative safety of BTX-A and methocarbamol. Future studies should incorporate a standardized and systematic assessment of adverse events.

## 5. Conclusions

Numerous studies have examined and compared the different treatments used for treating temporomandibular myofascial pain. However, a consensus regarding the optimal approach has yet to be reached, making the establishment of an ideal clinical guideline a difficult issue.

Both BTX-A and methocarbamol were associated with significant improvements in pain perception and maximum mouth opening, with no statistically significant between-group differences for these global outcomes. In an exploratory analysis, BTX-A was associated with a greater reduction in active trigger points in the right masseter and temporalis muscles, a finding that warrants confirmation in future studies.

BTX-A did not show statistically significant differences compared with methocarbamol in global outcomes, and in the absence of a placebo group, within-group improvements cannot be attributed solely to the active treatments.

This paper’s outcomes highlight the need for further studies to thoroughly investigate the underlying mechanisms and the duration of these therapeutic effects. Additionally, it is essential to determine the suitability of botulinum toxin type A (BTX-A) for various patient groups. It is also recommended that future research explore, through novel analyses, the role of oral anatomy in the symptoms and treatment of temporomandibular myofascial pain, as well as its potential relationship with sleep physiology and bruxism.

Future trials with adequate allocation concealment, blinded outcome assessment, and appropriate adjustment for multiple comparisons are needed to determine whether the side-specific differences in trigger point reduction observed in this exploratory analysis reflect a true treatment effect or a chance finding.

## Figures and Tables

**Table 1:** Baseline demographic and clinical characteristics of the study participants.

Characteristics		Control Group-Methocarbamol (*n* = 111)	Intervention Group-BTX-A (*n* = 124)	*p* Value
Age (years)		41.52 (11.34)	40.88 (12.01)	0.678 *
Sex *n* (%)	Female	92 (82.9%)	104 (83.9%)	0.842 **
	Male	19 (17.1%)	20 (16.1%)	
**Baseline Clinical Measures**				
VAS Scale (0–10)		7.44 (1.62)	7.54 (1.71)	0.651 *
PPI Index (0–5)		3.32 (1.05)	3.34 (1.16)	0.892 *
Mouth Opening (mm)		37.91 (4.52)	36.90 (5.76)	0.141 *
**Trigger Points per Muscle Site**				
Right Masseter		2.18 (0.90)	1.89 (1.11)	0.030 *
Left Masseter		1.90 (1.10)	1.92 (1.05)	0.889 *
Right Temporalis		1.86 (0.95)	1.60 (1.17)	0.068 *
Left Temporalis		1.68 (1.01)	1.48 (1.15)	0.165 *
Total Trigger Points		7.62 (2.81)	6.89 (3.34)	0.076 *

Data are presented as Mean (SD). *p*-Values were determined using * Student’s *t*-test for continuous variables and ** Chi-square test (*χ*^2^) for categorical variables.

**Table 2:** Comparison of active trigger points before and after treatment in both study groups.

Active Trigger Points	Control Group-Methocarbamol (*n* = 111)		Intervention Group-BTX-A (*n* = 124)		* *p*-Value (Interaction)
	Pre Treatment	Post Treatment	Pre Treatment	Post Treatment	
Right Masseter	2.18 (0.90)	1.66 (1.01)	1.89 (1.11)	1.37 (0.99)	0.028
Left Masseter	1.90 (1.10)	1.43 (1.01)	1.92 (1.05)	1.36 (1.02)	0.601
Right Temporalis	1.86 (0.95)	1.33 (0.98)	1.60 (1.17)	0.99 (0.58)	0.009
Left Temporalis	1.68 (1.01)	1.18 (0.88)	1.48 (1.15)	0.98 (0.43)	0.132

Data are presented as Mean (SD). * *p*-Values represent the Group × Time interaction effect calculated via a two-way mixed-design ANOVA on raw individual patient data. The reported F-test accounts for individual response trajectories and within-subject variance (right masseter: *F* = 4.88, *p* = 0.028; right temporalis: *F* = 6.95, *p* = 0.009).

**Table 3:** Comparison of overall pain intensity (VAS) and subjective Pain Perception Index (PPI) before and after treatment in both study groups.

Pain	Control Group-Methocarbamol (*n* = 111)		Intervention Group-BTX-A (*n* = 124)		* *p*-Value
	Pre Treatment	Post Treatment	Pre Treatment	Post Treatment	
VAS scale (0–10)	7.44 (1.62)	5.41 (2.75)	7.54 (1.71)	5.04 (2.62)	0.130
PPI (0–5)	3.32 (1.05)	2.35 (1.15)	3.34 (1.16)	2.08 (1.12)	0.071

Data are presented as Mean (SD). * *p*-Values represent the intergroup comparison at 5 weeks, calculated using Analysis of Covariance (ANCOVA) with the baseline (pre-treatment) score as the covariate.

**Table 4:** Comparison of oral opening assessment measured with Therabite before and after treatment in both study groups.

Oral Opening	Control Group-Methocarbamol (*n*= 111)		Intervention Group-BTX-A (*n* = 124)		* *p*-Value
	Pre Treatment	Post Treatment	Pre Treatment	Post Treatment	
Mouth Opening Measurement (TheraBite)	37.91 (4.52)	38.89 (3.28)	36.90 (5.76)	39.02 (3.03)	0.125

Data are presented as Mean (SD). * *p*-Value represents the intergroup comparison at 5 weeks, calculated using Analysis of Covariance (ANCOVA) with the baseline (pre-treatment) mouth opening as the covariate.

## Data Availability

The original contributions presented in this study are included in the article. Further inquiries can be directed to the corresponding author(s).
